# International beeswax trade facilitates small hive beetle invasions

**DOI:** 10.1038/s41598-019-47107-6

**Published:** 2019-07-23

**Authors:** Franck Ouessou Idrissou, Qiang Huang, Orlando Yañez, Peter Neumann

**Affiliations:** 1 Institute of Bee Health, Vetsuisse Faculty, University of Bern, Bern, Switzerland; 2 Agroscope, Swiss Bee Research Centre, Bern, Switzerland

**Keywords:** Invasive species, Haplotypes

## Abstract

International trade can facilitate biological invasions, but the possible role of beeswax trade for small hive beetles (SHBs), *Aethina tumida* Murray (Coleoptera: Nitidulidae) is poorly understood. SHBs are parasites of social bee colonies native to sub-Saharan Africa and have become an invasive species. Since 1996, SHBs have established in all continents except Antarctica. Here, we combine mitochondrial DNA analyses (*COI* gene, N = 296 SHBs, 98 locations) with previously published beeswax trade data (FAO) for 12 confirmed SHB invasions. Our genetic data confirm previous findings and suggest novel SHB African origins. In nine out of 12 invasion cases, the genetic and beeswax trade data match. When excluding one confirmed pathway (bee imports) and two cases, for which no FAO data were available, the genetics and beeswax trade data consistently predict the same source. This strongly suggests that beeswax imports from Ethiopia, South Africa, Tanzania and the USA, respectively, have mainly been responsible for the past invasion success of this beetle species. Adequate mitigation measures should be applied to limit this key role of beeswax imports for the further spread of SHBs. Combining genetics with trade data appears to be a powerful tool to better understand and eventually mitigate biological invasions.

## Introduction

Invasive species are a major threat to food security and conservation of natural biodiversity^1,2^. Typically, such biological invasions follow a jump dispersal pattern reflecting human assisted transmission often across the entire globe^3,4^. In particular, there is growing evidence that international trade can play a major role in spreading alien species^5,6^, but in many cases the actual trading routes and goods involved remain poorly understood. Aiming at mitigating the global impact of invasive species, a better understanding of the role of specific international trading routes as transmission pathways appears therefore crucial. Since genetic tools are routinely used to reconstruct routes of invasion^7^, one feasible approach seems to be a combination of genetics data with public data on international trade^8^ for specific cases of invasive species. This also holds true for invasive pests and pathogens associated with managed honey bees, *Apis mellifera* Linnaeus (Hymenoptera: Apidae)^9^.

One invasive species associated with honey bees is the small hive beetle (=SHBs), *Aethina tumida* Murray (Coleoptera: Nitidulidae)^10^. SHBs are parasites of honey bee colonies native to sub-Saharan Africa^11,12^. Larval and adult SHBs feed on honey, pollen, bee larvae, as well as dead or live adult bees^13–15^. SHB larvae can cause severe damage to honey bee colonies^14^, often resulting in the full structural collapse of the entire nest^12^. Since 1996, SHBs have emerged as an invasive species and have now established on all continents except Antarctica^10,16–18^. At present, it is still unclear whether a single or multiple introductions into the USA have occurred^19–21^. SHBs in Australia appear to have a different origin than beetles in North America and the initial North American beetles shared the same source^21^. The outbreaks in Quebec (Canada)^22^ appear to have originated from the USA^10,21,22^ and the SHBs confirmed in Alberta (Canada) in 2006^22^ were probably a novel introduction via Australian package bees^21^. SHBs confirmed in the Calabria region (Italy) in 2014^23^ appear to have been introduced from Cameroon^24^. This introduction into Calabria was followed by man-mediated migration to Sicily^24^. SHBs detected and originally reported to be widespread in Egypt^25^ were not confirmed by a latter extensive survey^26^, thereby suggesting a possible false positive diagnosis in the first place. In 2004, SHB larvae were intercepted in a shipment of queens from the USA to Portugal, but pest establishment was successfully prevented by rigorous sanitation^27^. Recently, introductions into Hawaii (2010)^10^ and Brazil (2015)^16^, were traced back to South Africa^28^ and the USA^29^, respectively. None of the confirmed SHB introductions reported in Portugal (2004)^27^, Jamaica (2005)^10^, Mexico (2007)^10^, Cuba (2012)^10^, El Salvador (2013)^10^, Nicaragua (2014)^10^, Philippines (2014)^10^, South Korea (2016)^17^ and Mauritius (2016)^18^ have so far been studied genetically.

In its new ranges, SHBs can have a strong impact on local honey bees and can also infest colonies of other social bees^10^ as well as nests of solitary bees^30^. It appears therefore high time to slow down the ongoing spread of SHBs until better mitigating options will become available^31^. Central to that is clearly the identification of the actual transmission means. So far, a whole range of actual and possible transmission pathways has been identified, with import of bees and bee products being the major confirmed contributors so far^10^. Accordingly, countries have put in place legislation and quarantine measures to reduced chances for entry of SHBs as well as other bee pests and diseases^9^. While most of these efforts have focused on the control of living bee imports, hive products have so far been rather neglected, even though one introduction of SHBs into Canada could be traced back to beeswax imports^22^. Therefore, it seems obvious that at least some additional SHB introductions may have occurred in association with international trade of beeswax.

Beeswax is a creamy coloured substance used by worker bees to build the comb that forms the structure of their nest^32^. It is widely used by humans, e.g. in cosmetics, pharmaceutical preparations and food production^33^. In particular, beekeepers use large quantities of beeswax for making beeswax comb foundation^32,33^. Beeswax can significantly range in quality^34^ and respective international trade can occur in various forms, ranging from non-processed “crude” wax over wax foundations to highly processed ones (i.e. melted and purified)^9,32–35^. For beeswax in the form of “honeycomb”, there are trade regulations with respect to SHBs available^36^. Either the beeswax originates from a country free of *A. tumida* infestation or precautions, such as “thoroughly” cleaning, have been taken to prevent infestation/contamination with SHB life stages^9,36^. However, for processed beeswax there are currently no trade restrictions with respect to SHBs^9,36^, probably because wax processing is assumed to kill all SHB life stages (e.g. melting). Interestingly, countries in sub-Saharan Africa, the endemic range of SHBs^10,12^, only participate in international trade with the sale of beeswax and honey^8^, the latter not being confirmed as a SHB transport means so far^10^. Nevertheless, correlations between global beeswax trade routes^8^ and confirmed SHB introductions with respective areas of origin have yet to be taken into consideration. This is mainly due to few data sets existing on molecular markers tracing back the origin and possible transmission pathways of SHBs^19–21,24,28,29^. These previous studies could obviously not have covered all confirmed introductions so far and/or only considered comparatively few native locations of SHBs.

The aim of this study is to shed further light on possible SHB invasion pathways to mitigate its further spread. For that purpose, we here combine for the first time genetic data of 12 confirmed SHB invasions with FAO data on international trade of beeswax^8^. If the latter constitutes a major means of SHB transport globally, we suspect that in the majority of cases genetic traces of origin match with respective data on the imports of beeswax.

## Results

### Mitochondrial *COI* haplotypes

Analysis of mitochondrial *COI* DNA sequence data from 296 individuals revealed 90 unique haplotypes representing 0.857 ± 0.018 and 0.02280 ± 0.00103 (mean ± SD) haplotype and nucleotide diversity, respectively. Among the sequences analysed, 118 segregating (polymorphic) sites were detected (Table 1), 89 of which were parsimony-informative and 29 were singleton sites.

**Table 1:** Number of analysed individuals (N), polymorphic sites (S), parsimony informative sites (PIS), number of haplotypes (h), haplotype diversity (Hd), nucleotide diversity (pi) and average number of differences (K) in mt-DNA sequences computed for *Aethina tumida* collected from five geographical regions.

Regions	Sample size (N)	Polymorphic sites (S)	Parsimony informative sites (PIS)	Number of haplotypes (h)	Haplotype diversity (Hd)	Nucleotide diversity (pi)	Average number of nucleotide differences (K)
Australia	30	19	10	4	0.510 ± 0.109	0.00285 ± 0.00076	2.522
Americas	115	16	13	5	0.301 ± 0.056	0.00183 ± 0.00039	1.518
Europe	10	45	43	4	0.889 ± 0.075	0.02070 ± 0.00256	20.289
Asia	21	36	36	2	0.381 ± 0.101	0.01404 ± 0.00370	13.714
Africa	120	105	73	80	0.983 ± 0.005	0.02490 ± 0.00148	13.646
All	296	118	89	90	0.857 ± 0.018	0.02280 ± 0.00103	12.450

**Figure 1:**
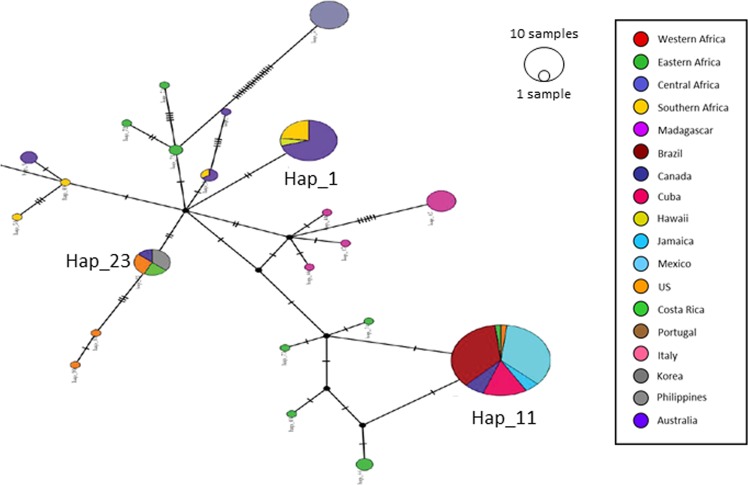
Mitochondrial haplotype network constructed from samples of *Aethina tumida* (N = 296) using the TCS method as implemented in PopArt. The areas of the circles are proportional to the number of samples sharing each haplotype. Small ticks on branches indicate the number of mutations separating haplotypes. Each colour represents haplotypes found in a region.

81 haplotypes were found in a single country (Australia, Benin, Burkina-Faso, Burundi, DR Congo, Ethiopia, Italy, Kenya, Madagascar, Malawi, Nigeria, Central African Republic, South-Sudan, Sudan, Tanzania, Togo, Uganda, and the USA) while the nine others were present in at least two different countries. The most widely distributed haplotype (Hap_11) was found with a global frequency of 35.1% and was detected in Africa (Tanzania) and the Americas (Brazil, Canada, Cuba, Jamaica, Mexico, and USA) (Fig. 1). The two other most frequent haplotypes were Hap_1 and Hap_23 with global frequencies of 11.5 and 4.7%, respectively. Whereas Hap_1 occurred in three countries (South Africa, Hawaii and Australia), Hap_23 was found in four countries (Canada, Costa Rica, USA, and South Korea) (Fig. 1).

The highest *COI* diversity was found in Africa with 80 out of 90 haplotypes present (*Hd* = 0.983 ± 0.005, *pi* = 0.02490 ± 0.00148, *K* = 13.6) (Table 1). Among the invaded areas, Europe (Portugal and Italy), where four haplotypes were detected, displayed the highest genetic diversity (*Hd* = 0.889 ± 0.075, *pi* = 0.02070 ± 0.00256, *K* = 20.3) (Table 1). The Americas, with five identified haplotypes, showed the lowest genetic diversity (*Hd* = 0.301 ± 0.056, *pi* = 0.00183 ± 0.00039, *K* = 1.518). From the five detected haplotypes in the Americas, four were found in the USA.

### Phylogenetic reconstruction

**Figure 2:**
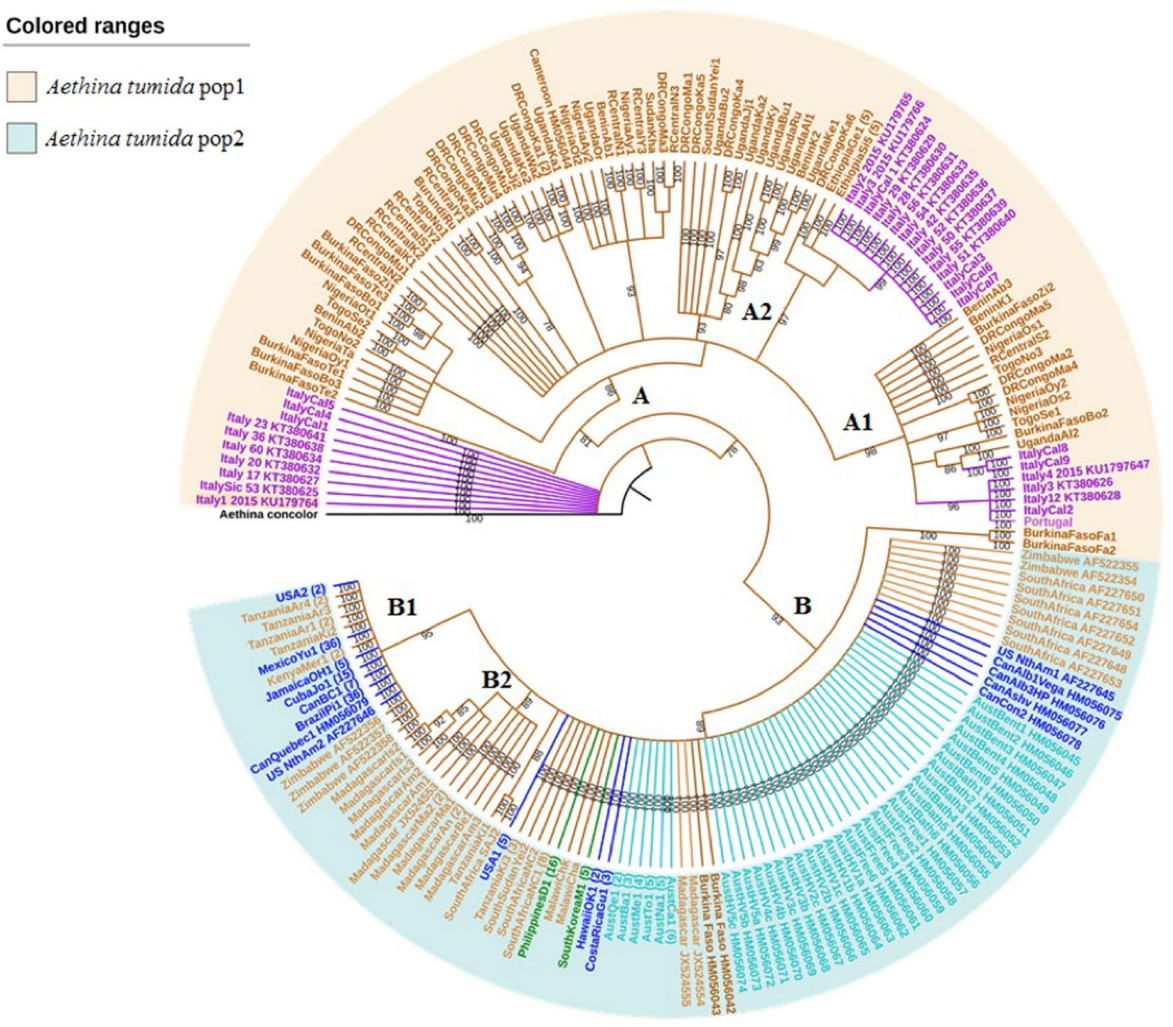
Bayesian phylogenetic tree of the *COI* gene showing relationships among *Aethina tumida* samples from Africa, the Americas, Asia, Australia and Europe. Posterior probabilities are shown above nodes with over 70% support. The different letters represent main clades (A, A1, A2, B, B1, B2). Each branch and label colour shows *A. tumida* samples collected from the same country and/or continent. Each colour range shows the main African population Pop1 (=beige) or Pop2 (=frost blue). *A. concolor* was used as outgroup.

The Bayesian phylogenetic reconstruction based on *COI* sequences separates the samples into six different clades, labelled A, A1, A2, B, B1, and B2 (Fig. 2). These different clades can be grouped in two main African populations labelled Pop1 and Pop2 (Fig. 2). Pop1 is consisted of Western (Benin, Nigeria, Togo, and Burkina Faso) and Central African (Cameroon, Central African Republic, and Democratic Republic of Congo) populations, but also some Eastern regions (Uganda, South Sudan, Sudan, Burundi, and Ethiopia). Pop2 includes some Eastern (Tanzania, Kenya, South Sudan, and Madagascar), Southern (South Africa, Malawi, and Zimbabwe) and Western African (Burkina Faso) populations. Pop2 shows 93% similarity with Burkina Faso samples from Fada-Ngourma. In Pop1 and Pop2 different clades are well-supported by posterior probabilities (>90%).

Most of the Italian specimens belong to clade A, mainly A1 and A2. The other Italian specimens, which are not included in clade A, show similarity with each other. In clade A1, the Italian samples (ItalyCal8, and ItalyCal9) cluster with one Ugandan sample (UgandaAl2) with a Bayesian posterior probability of 86%. In the same clade, other Italian samples (ItalyCal2, Italy12, Italy3, Italy4 2015) show a high similarity with the Portuguese sample (96%). In clade A2, Italian samples, which contains the first detected specimen in Calabria (ItalyCal1_KT380624), cluster with Ethiopian samples with a Bayesian posterior probability of 97%.

Clade B1 includes SHB from the USA (USA2, US NthAm2), Mexico, Jamaica, Cuba, Canada (British Columbia, Quebec) and Brazil. Interestingly, within this clade all samples cluster with Kenyan, Zimbabwean, and Tanzanian samples with a Bayesian posterior probability of 95%. In clade B, all samples from Australia, USA (USA1, USNthAm1), Canada (Alberta, Ashville, and Concordia), Costa Rica, Hawaii and South Korea show similarity with Tanzanian and South African samples.

### FAO data

We considered beeswax imports^8^ from countries with known native or introduced SHB populations up to two years before the introduction into new ranges (Table 2). Based on prior invasion history (USA, Australia)^10,15,22^, this appears to be a suitable time window. SHB populations probably have to increase first to cause clinical symptoms of infestation^37^, thereby fostering their detection by beekeepers.

**Table 2:** Combined genetics data (this study) and beeswax trade data (FAO)^8^ for 12 confirmed small hive beetle (SHB) invasions.

Invasion	Genetics	Beeswax	Origin
USA (mainland)	Kenya, Tanzania, Zimbabwe, South Africa	Tanzania	***Tanzania***
Mexico	Kenya, Tanzania, Zimbabwe	USA, Germany (no SHBs)	***USA***
Jamaica	Kenya, Tanzania, Zimbabwe	USA	***USA***
Cuba	Kenya, Tanzania, Zimbabwe	—	USA
Canada	Kenya, Tanzania, Zimbabwe, South Africa	USA	***USA***
Brazil	Kenya, Tanzania, Zimbabwe	USA, India (no SHBs)	***USA***
Costa Rica	Tanzania, South Africa	USA	***USA***
USA (Hawaii)	Tanzania, South Africa	—	USA, South Africa
South Korea	South Africa, Tanzania	USA	***USA***
Australia	South Africa, Tanzania	South Africa, China (no SHBs)	***South Africa***
Portugal	Unknown (same as Italy)	Spain, Germany, UK, France, Netherlands (all no SHBs)	USA
Italy	Unknown, Ethiopia, Uganda	Various countries, incl. Ethiopia	***Ethiopia***

The country of SHB Invasion, the possible country of origin in the endemic range in Africa (=Genetics), the country of origin for imported beeswax (=Beeswax) and the most parsimonious country of SHB origin (=Origin) are shown (−= no data available). In nine out of 12 invasion cases, the genetics and beeswax data match as indicated in ***bold***. For the Portugal case, import of queen bees was shown^35^ and for two further cases, beeswax trade data are not available (−= lack of FAO data^8^).

#### Beeswax imports into the Americas

From 1994 to 1996, Tanzania was the first African beeswax trade partner to the USA. The USA imported 39, 29 and 70 tons (=T) in 1994, 1995 and 1996 respectively. From 2000 to 2002, Canada imported 363.84 T of beeswax per year, of which ∼87% was from the USA. During this period, Canada did not import any beeswax from Africa. In the suspected period of introduction of SHBs into Jamaica (i.e. 2003–2005), FAO data clearly show that this country imported 3 T of beeswax only in 2003 and exclusively from the USA. From 2005 to 2007, the USA and Germany (no confirmed SHB cases) were the main beeswax suppliers of Mexico with an average of 81.33 and 75.33 T per year respectively (with a maximum import of 108 and 78 T in 2007). Costa Rica is a small beeswax importer with an average of 3.33 T per year from 2012 to 2014. During this period, Costa Rica imported only from two countries: Argentina (2 T; no confirmed SHB cases) and USA (1.33 T). In 2012 and 2013, Costa Rica imported beeswax exclusively from the USA with an average of 1 T per year. The USA, after India (no confirmed SHB cases), was the second largest beeswax exporter to Brazil with an average of 5.67 T per year from 2013 to 2015. There are no data available on the quantity of beeswax imported to Cuba.

#### Beeswax imports into Australia

For the period 1999–2001, data are not available in the FAO database. However, from 2005 to 2016 South Africa was the second largest beeswax supplier (after China) to Australia with an average of 80.6 T per year.

#### Beeswax imports into Italy, Portugal and South Korea

From 2012 to 2014, Italy imported from Asia (283.33 T), Europe (242.01 T), Africa (20 T) and the Americas (2 T). In 2012, Italy imported beeswax from Africa exclusively from Ethiopia (18 T). During this period, Italy did not import any beeswax from Cameroon. From 2002 to 2004, Portugal exclusively imported from Europe: Spain (20 T), Germany (5.67 T), UK (3.67 T), France (0.33 T) and Netherlands (0.33 T). None of these countries had any confirmed SHB cases. Portugal did not import any beeswax from the USA. South Korea imported beeswax from Americas exclusively from the USA with 11 and 16 T in 2015 and 2016 respectively.

#### Combination of genetics and beeswax trade data

**Figure 3:**
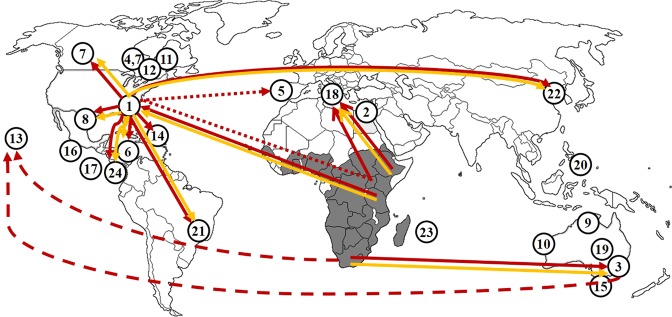
Small hive beetle native range, confirmed global introductions (up to May 2019) and invasion pathways. Please refer to Neumann *et al*.^10^ for further references. Endemic distribution range in sub-Saharan Africa (=dark grey areas), introductions (=white circles) and most likely invasion routes (arrows) are shown: (1) 1996, Charleston, South Carolina, USA, (2) 2000, Itay-Al-Baroud, Egypt, (3) 2001, Richmond, NSW, Australia, (4) 2002, Manitoba, Canada, (5) 2004, Lisbon, Portugal, (6) 2005, Jamaica (2010), (7) 2006, Alberta and Manitoba, Canada, (8) 2007, Coahuila, Mexico, (9) 2007, Kununurra, North Australia, (10) 2008, Perth Australia, (11) 2008, 2009, Quebec, Canada, (12) 2008, 2013 Ontario, Canada, (13) 2010, Pana’ewa, Big Island, Hawaii, (14) 2012, Cuba, (15) 2012, Naracoorte in Eastern South Australia; (16) 2013, El Salvador, (17) 2014, Nicaragua), (18) Sovereto, Calabria, Italy, (19) 2014, Renmark, Australia, (20) 2014, Lupon, Philippines, (21) 2015, Piracicaba, São Paulo State, Brazil^16^, (22) 2016, Miryang-si, GN, Korea^17^, (23) 2016, Mauritius island^18^. (24) 2016, Guanacoste, Costa-Rica (this study); (red line = our genetic data; solid red lines = invasion pathways suggested by genetics; dashed red lines = origin from Australia and/or South Africa; dotted red line = confirmed introduction from the USA via bee import^7^, but our data suggest the same African origin as for Italy; yellow line = matching beeswax trade data^8^ with our genetic data).

To shed light on the role of beeswax imports for the spread of SHBs, we combined our genetic data with beeswax trade data (FAO)^8^ for 12 confirmed SHB invasions. For that purpose, we considered imports from countries in the endemic range of SHBs and with prior confirmed cases^10,15–18,22^. In nine out of 12 invasion cases, the genetics and beeswax data matched, i.e. countries of origin suggested by genetics data matched with beeswax trade records (Table 2, Fig. 3).

## Discussion

Our genetic data combined with FAO data strongly suggest that imports of beeswax play a previously underestimated key role in propagating SHBs globally. Indeed, in nine out of 12 cases, beeswax trade data and our genetic data match. When excluding one confirmed introduction pathway (bee imports, Portugal^27^) and the two cases, for which no FAO data were available, the genetics and FAO trade data consistently predict the same source of SHBs. This creates an urgent demand to better consider the role of international beeswax trade for the spread of this invasive species.

The results confirm the African origin of this invasive species^10,12,15^ and that SHBs found in Australia and Canada are most likely originating from South Africa and USA respectively^21^ (Fig. 3). Similarly, our genetic data are also in line with recent reports on the origin of SHBs in Hawaii^28^ and Brazil^29^, suggesting that SHBs discovered in Hawaii originated from South Africa and those found in Brazil came from the USA. Tanzania, South Africa, Ethiopia and Uganda have been identified as sources of invasive populations respectively found in the USA, Australia and Italy. The data further suggest that the USA is the most probably source of spread of SHBs in Cuba, Jamaica, Costa Rica, Mexico and South Korea. The samples from Costa Rica were here confirmed to be *A. tumida*.

The highest *COI* diversity was found in Africa with 80 out of 90 identified mitochondrial haplotypes. This high genetic diversity confirms previous findings^21^ as well as the African origin of SHBs. Furthermore, the phylogenetic reconstruction suggests the existence of at least two large African populations, in which several haplotypes are present. The population Pop1 is more heterogeneous than population Pop2, thereby suggesting that the large region (West, Central and Eastern Africa) is probably the SHBs nucleus of origin. The comparatively high mobility of adult SHBs^10^ and resulting panmixis^21^ could explain the non-grouping of the different sub-populations by country in the phylogenetic tree. In addition, Pop2 showed a considerable similarity with samples from Burkina Faso (Fada-Ngourma) belonging to Pop1. This population may have invaded the southern and eastern regions. This could explain the homogeneity and low diversity observed within population Pop2. Based on our data, it is not clear if these two SHB populations actually overlap or not. A more exhaustive sampling in the neighbouring geographic areas between the two populations and more genetic markers will be required to shed light on the causes for this observed divergence.

In the invaded areas (Americas, Australia and Asia) the *COI* diversity was low when compared to Africa, which is a logic consequence of genetic bottlenecks due to invasion events^38^. Among these areas, the Americas showed the lowest genetic diversity probably reflecting comparatively few introduction events^21^. Alternatively, but not mutually exclusive, an original genetic diversity may have been lost secondarily.

Our results clearly show that one of the haplotypes (Hap_11) present in the Americas (USA, Brazil, Canada, Cuba, Jamaica and Mexico) is the same as that found in Tanzania. Combining our genetics data with the FAO data base^8^, the most likely invasion scenario is that the first specimens found in South Carolina in November 1996^15^ were introduced via infested beeswax containers from Tanzania. Since it can take years for mass reproduction with obvious clinical symptoms to occur^15^, SHBs could have easily been introduced one to two years before their confirmation. Indeed, the USA imported 39 and 29 T of beeswax respectively in 1994 and 1996 from Tanzania^8^. These SHBs almost certainly crossed the USA border to invade Quebec in 2008 (<25 km)^22^.

Our data do not confirm the previous finding of an Australian origin of Canadian SHBs^21^. Most likely, the SHBs introduced from Australia back then were not able to establish a local population in their northern distribution limit in Canada (but see Essex county, Ontario for an exception)^10^. It appears therefore most likely that novel introductions from the USA have occurred in the meantime. Similarly, according to our data, the USA is the most probable source of SHBs in other countries of the Americas (Brazil, Cuba, Jamaica and Mexico), which is also reflected in respective beeswax trade activities^8^.

The results of the haplotype analysis show that the second haplotype (Hap_23) found in the USA is also present in Canada, Costa Rica and South Korea. According to FAO statistics, Costa Rica and South Korea have not imported beeswax from Africa^8^. However, both countries have mostly imported beeswax from the USA^8^, thereby supporting that the SHBs detected in Costa Rica and more recently in South Korea originated from the USA. Alternatively, but not mutually exclusive, the SHBs in Costa Rica simply reflect the ongoing SHB invasion front in Central America^10^.

Our results confirm previous findings that SHBs found in Australia are most likely originating from South Africa^21^. Interestingly, the SHBs found in Hawaii are also similar to South African haplotypes^25^, thereby implying that Australian and Hawaiian beetles both originated from the same African source population. Alternatively, but not mutually exclusive, the Hawaiian SHBs may have originated from the Australian populations. Unfortunately, there are no FAO data available concerning beeswax imports from South Africa to Australia and to Hawaii, respectively.

When compared to the Americas, SHB populations found in Europe show a higher haplotype diversity, thereby supporting multiple introduction events^24^. Phylogenetic analysis shows that specimens from Italy belong to a different clade than the one in the USA and Australia. Our data therefore support earlier results that the occurrence of SHB in Italy is due to an independent introduction from Africa and not from the USA or Australia^24^. The phylogenetic tree clearly shows two clades, A1 and A2, to which most SHB specimen from Italy belong, therefore, suggesting two separate introductions into the Calabria region. The first specimen detected in September 2014 in Gioia Tauro in the Calabria region^23^ most likely originated from Ethiopia (clade A2). A second introduction into the same region most likely originated from Uganda (clade A1). Ethiopia and Uganda are among the largest producers of beeswax with respective productions of 5,542 and 1,308 T in 2016^8^. Therefore, SHBs detected in the region of Calabria (only a few kilometres from the port of Gioia Tauro) most probably came from infested wax containers from Ethiopia and/or Uganda. Interestingly, Italy however only imported beeswax from Ethiopia until 2012^8^. This suggests that SHBs were probably present in Calabria at least two years before their official confirmation similar to the USA and Australia^15^. Moreover, it also shows that those SHBs in Calabria most likely originating from Uganda must have arrived via another yet unidentified pathway, e.g. bee imports as in the case of Portugal 2004^27^. Fascinatingly, the Portuguese sample shows a very high similarity (96%) with some Italian samples from Calabria, thereby strongly suggesting the same yet unidentified African origin (assuming that SHBs were not present in Italy back in 2004). The FAO data show that beeswax was not imported into Portugal from any country with known SHB populations^8^. Indeed, the *A. tumida* larvae were detected in a shipment of queens from Texas (USA)^27^. Since the Portuguese sample therefore unequivocally originated from the USA, this strongly implies that at least a second, previously not detected, introduction into the USA must have occurred independently from the Tanzanian one (see above). Therefore, we can here clarify the question whether a single or multiple introductions into the USA have occurred^21^. Based on our genetic data, it seems safe to assume that at least two independent introductions into the USA must have occurred.

The combination of our genetic and beeswax trade data^8^ enabled light to be shed on a previously overlooked key factor for SHB spread. Since the origin of the Portugal SHB introduction in 2004 can unambiguously be traced back to shipment of queen bees from the USA^36^, only for 11 out of the 12 confirmed introductions, which were investigated here, sources could not certainly be traced back so far. Correlations are not causations, however, given that two independent data sets match in their correlations (genetics and FAO), this further supports a causal relationship. Indeed, in the nine remaining cases, for which both genetic and FAO data were available, both data sets consistently suggest the same country of origin. This strongly suggests that international beeswax trade is the major factor explaining the past invasion history of small hive beetles. It seems therefore most unfortunate that nobody paid really attention up to now on the role of beeswax for SHB spread. Interestingly, the international transport of beeswax usually occurs via ships^39^ and some SHB introductions so far occurred near major harbors (Charleston, USA^15^; Gioia Tauro, Italy^23^) or on islands (Jamaica, Hawaii, Cuba, Philippines)^10^. Since the international shipping industry is responsible for the carriage of ~90% of world trade^39^, it appears unsurprising that the surroundings of harbours were starting points of several SHB invasions. It seems therefore prudent to take into account beeswax trade for adequate mitigation measures to further limit the spread of this pest species.

Ideally, beeswax shipments are not contaminated prior to sending. However, this appears notoriously difficult in regions with SHB populations due to the highly mobile and cryptic behaviour of the adult beetles^10,40^. Regardless of the actual beeswax processing stage^33,34^, adult SHBs are therefore very likely to invade and successfully hide in any beeswax trade package. Indeed, even small commercial honey bee queen cages, which are obviously much easier to visually screen by the beekeepers compared to containers and large boxes, were already responsible for two independent SHB invasions (Portugal^27^; Northern Territory, Australia^10^). It therefore appears inevitable that adult SHBs can and eventually will invade any beeswax trade containers during packaging. Considering adult SHB survival under starvation (at least 12 days without any food and water; Peter Neumann, unpublished data), chances for adult SHB invasion of and hiding^39^ in beeswax trade containers as well as the impact of this invasion on bees in the new ranges, it seems as if beeswax imports from countries with known SHB populations should be banned. Alternatively, rigorous quarantine measures in the importing countries might help, but in light of our rather fragmentary knowledge of SHB physiology, this appears risky.

In conclusion, our genetic results (1) confirm the African origin of SHBs, (2) show a high genetic diversity in Africa, (3) suggest two main native populations, (4) confirm previous findings and (5) suggest novel origins. Since our genetic data match well with data on international trade of beeswax^8^, a closer investigation of international beeswax trade may enable to slow down the further spread of this invasive species. In general, it appears worthwhile to use this combination of genetics and public trade data in more cases of invasive species.

## Methods

### Sample collection

Adult SHBs (N = 1542) were manually sampled^41^ from 101 naturally infested *A. mellifera* colonies from 98 locations both in the native range of SHBs in sub-Saharan Africa (Benin, Togo, Nigeria, Burkina-Faso, Central African Republic, Democratic Republic of Congo, Uganda, Sudan, South Sudan, Ethiopia, Kenya, Tanzania, Malawi, Burundi, Madagascar and South Africa) as well as from 11 confirmed SHB introductions (USA, Australia, Canada, Philipines, Mexico, Jamaica, Hawaii, Cuba, Italy, Brazil and South Korea). Samples from Guanacoste, Costa Rica taken in 2016 by J. Pettis were also included because detailed morphometric analysis^41^ confirmed that the samples do belong to *A. tumida*. The last remaining SHB larvae from the Portugal introduction in 2004^27^ was also included. *Aethina concolor* specimen collected in Australia were used as outgroup. Please refer to Table 3 for details of the sampling. All samples were preserved in 70% Ethanol, transported at room temperature and stored at −20 °C in a laboratory until further analyses.

**Table 3:** Sampling overview.

Country	Site (N of sequenced individuals)	Year
Australia	Townsville (5), Cairns (6), Nambour (5), Victoria-Melbourne (4), Bathurst (3), S.E. Queensland (2)	2016
Benin	Ketu (3), Abomey (3)	2015
Brazil	Piracicaba (30), Sao Pedro (6)	2016
Burkina-Faso	Fada N’gourma (2), Tenkodogo (3), Ziniaré (2), Bobo Dioulasso (3)	2015
Burundi	Rusiga (1)	2016
Canada	Canary (6), Mckenzie Road (1), LeFeuvre Road (1), Eco-dairy (1)	2015
Central African Republic	Kelengô (2), Ndara (3), Sibut (2), Yeremon (3)	2015
Costa Rica	Guanacoste (3)	2016
Cuba	Jovenallos (5), San Nicolas de Bari (5), Jaruco (5)	2016
Democratic Republic of Congo	Maboya 1 (2), Maboya 2 (1), Maboya 3 (1), Maboya 4 (1), Mulo 1 (1), Mulo 2 (2), Mulo 3 (1), Mulo 4 (1), Kambo 1 (1), Kambo 2 (1), Kambo 3 (2), Kambo 4 (2)	2016
Ethiopia	Gedeo zone (5), Sidama zone (5)	2016
Italy	Candinoni (1), Rizziconi (1), Melicucco (1), Polistena (1), Feroleto Della Chiesa (1), Taurianova (1), Altilia (3)	2016
Jamaica	Old Habour (5)	2016
Kenya	Meru (2)	2016
South Korea	Miryang (5)	2017
Madagascar	Ambat (3), Anjozorobe (1), Beomby (2), Isatra (3), Mantasoa (3)	2016
Malawi	Chawala (1), Chakhuntha (1)	2015
Mexico	San Ignacio (4), Llera (4), Linares (4), Tancitaro (4), Carretera (4), Candelaria (4), Benito Juarez (4), Tepich (4), Yucatan (4)	2016
Nigeria	Osogbo (2), Ayangba (2), Otan (2), Oyan (2), Taraba (1)	2015
Philipines	Davao 2 (6), Davao 3 (5), Davao 4 (5)	2015
Portugal	Lisboa region (1 Larvae)	2004
Republic of South Africa	Northern Cape (9)	2016
South Sudan	Yei City (2)	2015
Sudan	Khartoum (1)	2015
Tanzania	Kilimanjaro (8), Arusha (6)	2016
Togo	Seva (2), Notsie (3)	2015
Uganda	Jjangano (2), Keirere (2), Rubona (1), Kyanyamutale (1), Oryang-Ojuma (1), Busiwu (2), Alik (2), Wasswa/Kavunza (2), Kanyonza (2)	2015
USA	Baton Rouge 1 (2), Baton Rouge 2 (3), Baton Rouge 3 (3), Oahu kunia (2)	2016

The country, site and year of sampling are shown as well as the number of sequenced individuals per site.

### Genomic DNA extraction

A modified protocol from Neumann et *al*.^41^ was used. In brief, all samples were crushed individually in 2 mL microcentrifuge tubes containing 5 mm metal beads and 100 µL TN buffer (10 mmol/L Tris, 10 mmol/L NaCl; pH 7.6). Crushed samples were homogenized with a TissueLyser (QIAGEN, Hombrechtikon, Switzerland) for 25 sec at 20 1/sec frequency using a Qiagen Retsch^®^MM 300 mixer mill (Thermo Fisher Scientific, Zurich, Switzerland) and centrifuged for 60 sec at 2000 rpm. 50 µL of supernatant was taken from the homogenate and used for DNA extraction using innuPrep DNA Mini Kit (Analytik Jena, Jena, Germany) by following the manufacturer’s recommendations. The DNA yield and purity of each sample were checked using a Spectrophotometer Thermo Scientific^TM^ Nanodrop 2000 (Axon lab, Baden-Dättwill, Switzerland).

### PCR amplification and COI gene sequencing

A fragment of Cytochrome C Oxidase subunit I (*COI*) gene was amplified using primers AT1904S (5′-GGTGGATCTTCAGTTGATTTAGC-3′) and AT2953A (5′-TCAGCTGGGGGATAAAATTG-3′) for SHB specimens^19^. Additionally, another *COI* fragment was also amplified with LCO1490 (5′-GGTCAACAAATCATAAAGATATTGG-3′) and HCO2198 (5′-TAAACTTCAGGGTGACCAAAAAATCA-3′) for *A. concolor* specimen^41^. PCR tests were carried out in 25 μL (15.88 μL millipore water, 5 μL 5x reaction buffer, 1 μL (0.4 μmol/L) each primer (reverse and forward), 0.125 μL (0.63 units) *Taq* DNA polymerase, 2 μL tenfold-diluted DNA) in a Biometra^®^ Thermal Cycler (Thermo Fisher Scientific, Switzerland) as follows: 95 °C for 2 min, 35 cycles of 95 °C for 20 sec, 56 °C for 30 sec, 72 °C for 30 sec, and 72 °C for 2 min. Positive and negative controls were included in each PCR (DNA of previously identified SHB specimen from Nigeria (based on both morphometrics and DNA sequence data) or millipore water). Aliquots of PCR products were run by electrophoresis on a 2% (w/v) agarose gel.

The amplicons were purified with ExoSAP-IT^TM^ (Thermo Fisher Scientific, Zürich, Switzerland) and sequenced in both directions (forward and reverse). For a better coverage of the 1091 bp fragment to be sequenced, two internal primers, Aet-int-F (5′-CTTCTGCTACAATAATTATTGC-3′) and Aet-int-R (5′-TTGTGTACCATGAAGAGTAGC-3′) were added to the sequencing reactions^42^. All samples were analyzed using the Big Dye^®^ Terminator v3.1 cycle sequencing kit (Thermo Fisher Scientific, Zurich, Switzerland). Sequencing was performed using a 96-capillary ABI PRISM^®^ 3730XL Genetic Analyzer (Thermo Fisher Scientific, Zurich, Switzerland). The nucleotide sequences were deposited in the GenBank database under accessions MK024945-MK025231.

### DNA sequences alignment and analyses

The sequencing data were assembled and edited with Sequencher^®^software v5.4.1 (Gene codes corporation, Michigan, USA). The consensus sequences obtained were aligned using the MEGA 7 package and the MUSCLE alignment algorithm^43^, and compared to reference nucleotide sequence available in the GenBank database (http://www.ncbi.nlm.nih.gov/genbank/). All sequences with bad quality or ambiguous electropherograms were sequenced one in each direction twice and completely removed if still unclear. All SHB COI sequences from non-sampled countries available in GenBank database were added for further analyses.

The alignment was performed on sequences of different lengths, ranging from 739 bp (*A. concolor*) to 980 bp (*A. tumida*). The program MrModelTest v2.3^28^ was used to test models of evolution on SHB *COI* sequences for Bayesian Inference analyses. The best-fit model of nucleotide substitution for all sequences was SYM + G selected by Akaike Information Criterion (AIC). Bayesian inference (BI) was implemented with MrBayes v3.2.6^44^. The evolutionary model employed six substitutions types (“nst = 6”), with the stationary state frequencies set to be equally fixed (“statefreqpr = fixed (equal)”). Rate variation across sites was modeled using a gamma distribution (“rates = gamma”). The Markov chain Monte Carlo (MCMC) search was run with 4 chains for 25,000,000 generations, with trees sampled every 200 generations and the first 6,250,000 trees discarded as ‘burn-in’. The 739 bp *COI* gene sequence from *A. concolor* was used as an outgroup to root the trees. ITOL (Iterative Tree of Life) v4.2 (https://itol.embl.de/) was used to visualize and annotate the phylogenetic tree.

Indices of sequence diversity, including number of mt-DNA haplotypes (*Nh*), haplotype diversity (*h*), nucleotide diversity (*pi*) and average number of pairwise nucleotide differences (*K*), were estimated using DnaSP v6^45^. These parameters were calculated for samples grouped into five geographical regions: (1) Africa; (2) Americas; (3) Australia; (4) Europe and (5) Asia. In addition, the genealogical and geographical relationships among *COI* haplotypes were analyzed using the TCS network as implemented in PopArt^46^.

### FAO data

Based on SHB invasion history, it can take two years after introductions before official pest confirmation (USA: 1996–1998)^15^. We therefore took advantage of published data^8^ to collect information about imports of beeswax into countries with recent introductions at least two years before the notification of the presence of the SHB.
